# Predicting Metabolic Syndrome With Machine Learning Models Using a Decision Tree Algorithm: Retrospective Cohort Study

**DOI:** 10.2196/17110

**Published:** 2020-03-23

**Authors:** Cheng-Sheng Yu, Yu-Jiun Lin, Chang-Hsien Lin, Sen-Te Wang, Shiyng-Yu Lin, Sanders H Lin, Jenny L Wu, Shy-Shin Chang

**Affiliations:** 1 Department of Family Medicine Taipei Medical University Hospital Taipei Taiwan; 2 Department of Family Medicine School of Medicine, College of Medicine Taipei Medical University Taipei Taiwan

**Keywords:** machine learning, decision tree, controlled attenuation parameter technology, metabolic syndrome

## Abstract

**Background:**

Metabolic syndrome is a cluster of disorders that significantly influence the development and deterioration of numerous diseases. FibroScan is an ultrasound device that was recently shown to predict metabolic syndrome with moderate accuracy. However, previous research regarding prediction of metabolic syndrome in subjects examined with FibroScan has been mainly based on conventional statistical models. Alternatively, machine learning, whereby a computer algorithm learns from prior experience, has better predictive performance over conventional statistical modeling.

**Objective:**

We aimed to evaluate the accuracy of different decision tree machine learning algorithms to predict the state of metabolic syndrome in self-paid health examination subjects who were examined with FibroScan.

**Methods:**

Multivariate logistic regression was conducted for every known risk factor of metabolic syndrome. Principal components analysis was used to visualize the distribution of metabolic syndrome patients. We further applied various statistical machine learning techniques to visualize and investigate the pattern and relationship between metabolic syndrome and several risk variables.

**Results:**

Obesity, serum glutamic-oxalocetic transaminase, serum glutamic pyruvic transaminase, controlled attenuation parameter score, and glycated hemoglobin emerged as significant risk factors in multivariate logistic regression. The area under the receiver operating characteristic curve values for classification and regression trees and for the random forest were 0.831 and 0.904, respectively.

**Conclusions:**

Machine learning technology facilitates the identification of metabolic syndrome in self-paid health examination subjects with high accuracy.

## Introduction

Metabolic syndrome is a cluster of disorders, including insulin resistance or hyperglycemia, visceral adiposity (identified by a large waistline or overweight), atherogenic dyslipidemia (eg, raised triglycerides or reduced high-density lipoprotein [LDL]), and endothelial dysfunction (characterized by elevated blood pressure) [1]. Metabolic syndrome has significant impacts on the development and deterioration of several diseases and is a critical predictor of cardiovascular diseases [2,3]. Numerous modifiable risk factors and practical intervention strategies regarding metabolic syndrome have been proposed [4-14]. Identifying high-risk patients to prevent the incidence and deterioration of metabolic dysregulation and relevant diseases is therefore vital.

A recent study showed that nonalcoholic fatty liver disease (NAFLD) is closely correlated to metabolic syndrome. Patients with metabolic syndrome frequently show an increase in fat accumulation in the liver (steatosis) and hepatic insulin resistance [15]. Nevertheless, the gold-standard method for NAFLD diagnosis is liver biopsy, which is a highly invasive procedure for patients. Several reports have demonstrated that ultrasound using FibroScan, also known as transient elastometry, can accurately assess the staging of NAFLD in a noninvasive manner with comparable results to liver biopsy [16,17]. The new models of FibroScan (marketed after 2013) can assess the staging of NAFLD using a liver stiffness score (E score) and a liver steatosis score (controlled attenuation parameter [CAP] score). Interestingly, the CAP score alone was found to be a useful indicator of the presence and severity of metabolic syndrome [18]. Using traditional statistical modeling, we previously validated this finding, confirming that the CAP score alone can be used to detect metabolic syndrome with moderate accuracy (area under the receiver operating characteristic curve [ROC] of 0.79), and the accuracy was improved to 0.88 when combined with other biomarkers [19].

Machine learning, whereby a computer algorithm learns from prior experience, was recently shown to have better performance over traditional statistical modeling approaches [20-22]. Various supervised machine learning models based on decision trees have been successfully applied to medical data [23-29] for accurate prediction of a wide range of clinical conditions such as myocardial infarction [30], atrial fibrillation [31], trauma [32], breast cancer [33-35], Alzheimer disease [36-38], cardiac surgery [27,39], and others [27,28,40-42]. However, each decision tree machine learning algorithm has its own strength and weakness. Therefore, comparing different decision tree algorithms can reduce the bias in the results and provide a more robust outcome. Accordingly, the aim of this study was to determine whether decision tree algorithms can predict the state of metabolic syndrome among self-paid health examination subjects who were examined with FibroScan.

## Methods

### Study Design

This was a single-center retrospective cohort study. The cohort comprised self-paid health examination subjects at the Health Management Center of Taipei Medical University Hospital who were examined with FibroScan from September 2015 to December 2018.

### Setting

The electronic healthcare records of subjects examined with FibroScan were reviewed at Taipei Medical University Hospital, which is a private, tertiary-care, 800-bed teaching hospital in Taiwan. The Institutional Review Board of Taipei Medical University Hospital approved the study design for data collection (TMU-JIRB No.: N201903080) in accordance with the original and amended Declaration of Helsinki. The requirement for informed consent was waived owing to the retrospective nature of the study.

### Population and Data Collection

The study included all Taiwanese adult patients aged >18 years who had undergone a self-paid health examination comprising an abdominal transient elastography inspection using FibroScan 502 Touch (Echosens, Paris, France). Individuals who underwent FibroScan examination on physician’s orders were excluded. The routine protocols of the Health Management Center were applied to all participants. The subjects were first interviewed by thoroughly trained personnel who verified the correctness of self-completed questionnaires on demographics, existing medical conditions, and medication use. In addition, the personnel confirmed adherence to health examination prerequisites (eg, overnight fasting for at least 8 hours) for the package chosen by the study participant. Those found to have not fulfilled the necessary prerequisites were advised to reschedule their appointment. Anthropometrics, including weight, height, waist circumference, and arterial pressure, were measured. Instruments were regularly calibrated per the manufacturer’s specifications. According to the chosen package, the required samples of blood, urine, and specimens were collected for laboratory tests. Regular laboratory test items included alpha-fetoprotein, glycated hemoglobin (HbA_1c_), serum glutamic oxaloacetic transaminase (GOT), serum glutamic pyruvic transaminase (GPT), uric acid, creatinine, blood urine nitrogen, red blood cell count, hemoglobin, hematocrit, mean corpuscular hemoglobin, mean corpuscular volume, mean corpuscular hemoglobin concentration, platelet count, white blood cell count, percentage of neutrophils, lymphocytes, monocytes, eosinophils and basophils, total protein, albumin, globulin, albumin/globulin ratio, total bilirubin, direct bilirubin, alkaline phosphatase, gamma-glutamyl transpeptidase (γ-GT), total cholesterol, LDL cholesterol, high-density lipoprotein (HDL) cholesterol, LDL/HDL ratio, triglycerides, fasting blood sugar, and thyroid-stimulating hormone. The estimated glomerular filtration rate (eGFR) was calculated using equations for the Modification of Diet in Renal Disease for Chinese patients [43], with chronic kidney disease (CKD) measured as follows: 175 × (Scr)^–1.234^ × (Age)^–0.179^ × 0.79 (if female). CKD was defined as an eGFR of <60 mL/min per 1.73 m^2^ of body surface (mL/min/1.73 m^2^), according to the definition from the Kidney Disease Outcomes Quality Initiative for CKD ≥ stage 3 [39,44]. Body mass index categories were defined as follows: obesity, ≥27 kg/m^2^; overweight, 24-26.9 kg/m^2^; and normal weight, <23.9 kg/m^2^, according to the ranges established for Asian populations by the Ministry of Health and Welfare of Taiwan [45].

### Outcome

According to the National Cholesterol Education Program Adult Treatment Panel III definition of metabolic syndrome consensus, metabolic syndrome was identified if at least three out of the following five symptoms were present: large waistline (80 cm for women and 90 cm for men), high triglycerides (150 mg/dL) or use of medication to control triglycerides, reduced HDL levels (<50 mg/dL for women and <40 mg/dL for men) or use of medication to control HDL, elevated blood pressure (systolic blood pressure 130 mmHg or diastolic blood pressure 85 mmHg) or use of relevant medication to control blood pressure, and increased fasting blood sugar (100 mg/dL) or use of relevant medication to control blood sugar. The classification of cutoff points was adopted from the National Cholesterol Education Program Adult Treatment Panel III definition consensus with ethnicity-specific cutoff points for waist circumference [46,47] and an equality principle for the five disorders.

### FibroScan

**Figure 1:**
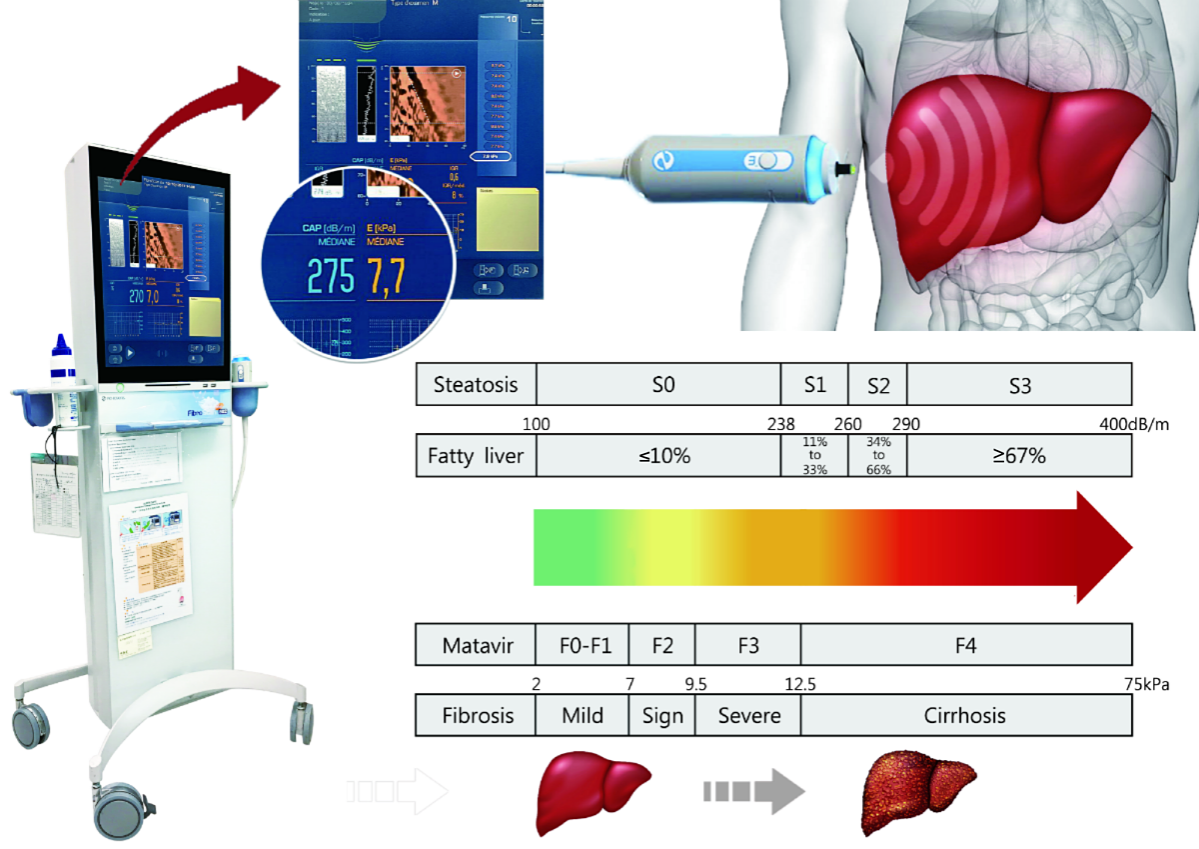
Illustration of the FibroScan device: liver diagnosis by ultrasound-based elastography. FibroScan measures fibrosis and steatosis in the liver. Measurements are performed by scanning the right liver lobe through the right intercostal space. The fibrosis result is measured in kiloPascals (kPa), and is normally between 2.5 and 6 kPa; the highest possible result is 75 kPa. Fibrosis score: F0 to F1, no liver scarring or mild liver scarring; F2, moderate liver scarring; F3, severe liver scarring; F4, advanced liver scarring (cirrhosis). The steatosis result is measured in decibels per meter (dB/m), and is normally between 100 and 400 dB/m. Steatosis can be graded from S0 to S3, corresponding to the severity of fatty liver from "0-10%" to "67% or more".

FibroScan is a noninvasive device that assesses the hardness of the liver using ultrasound-based elastography. Liver hardness is evaluated by measuring the velocity of a vibration wave, which is determined by measuring the time that the vibration wave takes to travel to a particular depth inside the liver from the skin (Figure 1). For each FibroScan inspection, two scores are reported: the CAP score and E score. The dashboard of FibroScan provides a CAP score only when an E score derived from identical signals is validated as successfully computed; higher E scores indicate higher transmission velocity and liver stiffness levels, and higher CAP scores indicate faster wave amplitude attenuation and higher levels of liver steatosis. Notably, the adoption of probe size (medium or extra large) is based on the recommendation of the instrumental autodetection function.

### Machine Learning Technique

#### Overview

A decision tree is a widely used effective nonparametric machine learning modeling technique for regression or classification purposes. To obtain solutions, a decision tree makes a sequential, hierarchical decision regarding outcome variables based on the predictor [48].

#### Classification and Regression Trees

Classification and regression trees (CART), the typical tree-based models, explore the structure of data, while evolving to visualize decision rules for predicting a categorical (classification tree) or continuous (regression tree) outcome [49]. The decision at each internal node is assessed by information gain or entropy to compare the value of attributes in the data from the root to each of the leaves. CART was generated through the “rpart” package in R [50].

#### C5.0

C5.0 is derived from C4.5 and ID3 with improvements according to the disadvantages of the predecessor trees. The “C50” package was applied to implement the C5.0 tree [51,52].

#### Chi-Square Automatic Interaction Detection

Chi-square automatic interaction detection (CHAID) is a specific decision tree using adjusted significance testing (Bonferroni testing) for prediction. An algorithm for recursive partitioning is implemented by maximizing the significance of a chi-square statistic for crosstabulations between the categorical dependent variable and the categorical predictors at each partition. Moreover, CHAID can create nonbinary trees since nominal, ordinal, and continuous data are used. CHAID tree is available from the “CHAID” package in R [53].

#### Conditional Interference Trees

Conditional inference trees (ctrees) embed tree-structured regression models into a well-defined theory of conditional inference procedures. They use a significance test procedure to select variables instead of selecting the variable that maximizes any information measure. In addition, ctree is applicable to all types of regression issues, including nominal, ordinal, numeric, censored, and multivariate response variables, as well as arbitrary measurement scales of covariates. A flexible and extensible computational tool in the “partykit” package of R is suitable for fitting and visualizing ctrees [54,55].

#### Evolutionary Learning of Globally Optimal Trees

Evolutionary learning of globally optimal trees (evtree) describes recursive partitioning methods that create models using a forward stepwise search. An evtree is learned using an evolutionary algorithm. Notably, a set of trees is initialized with random split rules in the root nodes. Mutation and crossover operators are then applied to modify the tree’s structure and tests that are applied in the internal nodes. After each modification step, a survivor selection mechanism identifies the best candidate models for the next iteration, terminating when the quality of the best trees ceases to improve. The “evtree” package in R applies an evolutionary algorithm for learning globally optimal classification and regression trees [56].

#### Generalized Linear Model Trees

Generalized linear model trees (glmtree) involve model-based recursive partitioning based on generalized linear models. They are convenient for fitting model-based recursive partitions using “mob” functions in R. A glmtree internally sets up a model-fit function for mob using the negative log likelihood as the objective function. It is also implemented by the “partykit” package in R [54,57].

#### Random Forest

Random decision forests are an ensemble learning method for classification, regression or other applications based on decision tree structures at the time of training. The idea of random forest is to create multiple decision trees (CART) and then combine the output generated by each of the decision trees. In the decision tree algorithm, the Gini index is a measure of the frequency of a randomly chosen element from the set that would be incorrectly labeled. The Gini index is calculated by subtracting the sum of the squared probabilities of each class from 1. This approach removes the bias that a decision tree model might introduce to a system while considerably improving the predictive power. In addition, random forests can be used to rank the importance of variables in a regression or classification problem in a natural manner, which can be conducted in the R package “randomForest” [58].

### Statistical Analysis

#### Basic Statistics

Statistical analysis was conducted using R (version 3.6.1; R Foundation for Statistical Computing, Vienna, Austria) or SPSS (version 17.0; SPSS Inc, Chicago, IL, USA) software.

Categorical variables were tested using the chi-square test or Fisher exact test. The nonparametric Mann-Whitney U-test was applied to determine differences in the median of continuous variables between the two groups. Multivariate logistic regression was employed to assess the significance of clinical data, and the variance inflation factor was also used to check for multicollinearity. *P*<.05 was considered statistically significant [59,60].

#### Principal Components Analysis

High-dimension data were processed by principal components analysis (PCA), using an orthogonal transformation to convert a set of observations of correlated variables to provide a two-dimensional or three-dimensional visualization with its leading principal components.

#### Receiver Operating Characteristic Curve

ROC curves were used to illustrate the diagnostic ability of classification trees in the machine learning methodology. The area under the ROC curve (AUC), true positive rate (also called sensitivity or recall), and false positive rate (specificity) are represented in a graphical plot [61]. The F1 score, which constitutes the harmonic mean of precision and recall, was also evaluated. The F1 score has been widely used in the natural language processing literature and for machine learning [62,63].

#### Missing Values

Data with missing values were statistically regulated by the expectation-maximization algorithm, which is an iterative procedure that preserves the relationship with other variables. Only 9 factors had missing values, and most of them accounted for less than 5% of the sample size. Direct bilirubin, which had the largest proportion of missing values (298/1333, 22.36%), was at high risk of multicollinearity; thus, it was not a crucial element in the model [64].

### Comparison of Decision Trees

To compare the performance of the aforementioned decision trees, the same setting for the training set and testing set was considered. In addition, the boundary for each tree’s height was limited between 4 and 5 instead of pruning each decision tree according to its own criteria. Finally, outcomes from each decision tree were summarized to investigate common and reliable results supporting the conclusions.

## Results

After data cleaning, a total of 1333 individuals undergoing self-paid annual health examination were enrolled in this study. The baseline characteristics of the 193 patients diagnosed with metabolic syndrome and 1140 participants without metabolic syndrome are compared in Table 1. All categorical elements were found to be extremely significant in the chi-square test. Among the continuous variables, most of the risk factors were highly significantly different between groups in the nonparametric test, although not enough evidence was found for age, alpha-fetoprotein, bilirubin, and thyroid stimulating hormone to support rejection of the null hypothesis. However, large samples and *P* value problems had to be considered owing to the numerous and complex data in this analysis [65]. The foremost factors were then validated by a series of additional evaluations.

**Figure 2:**
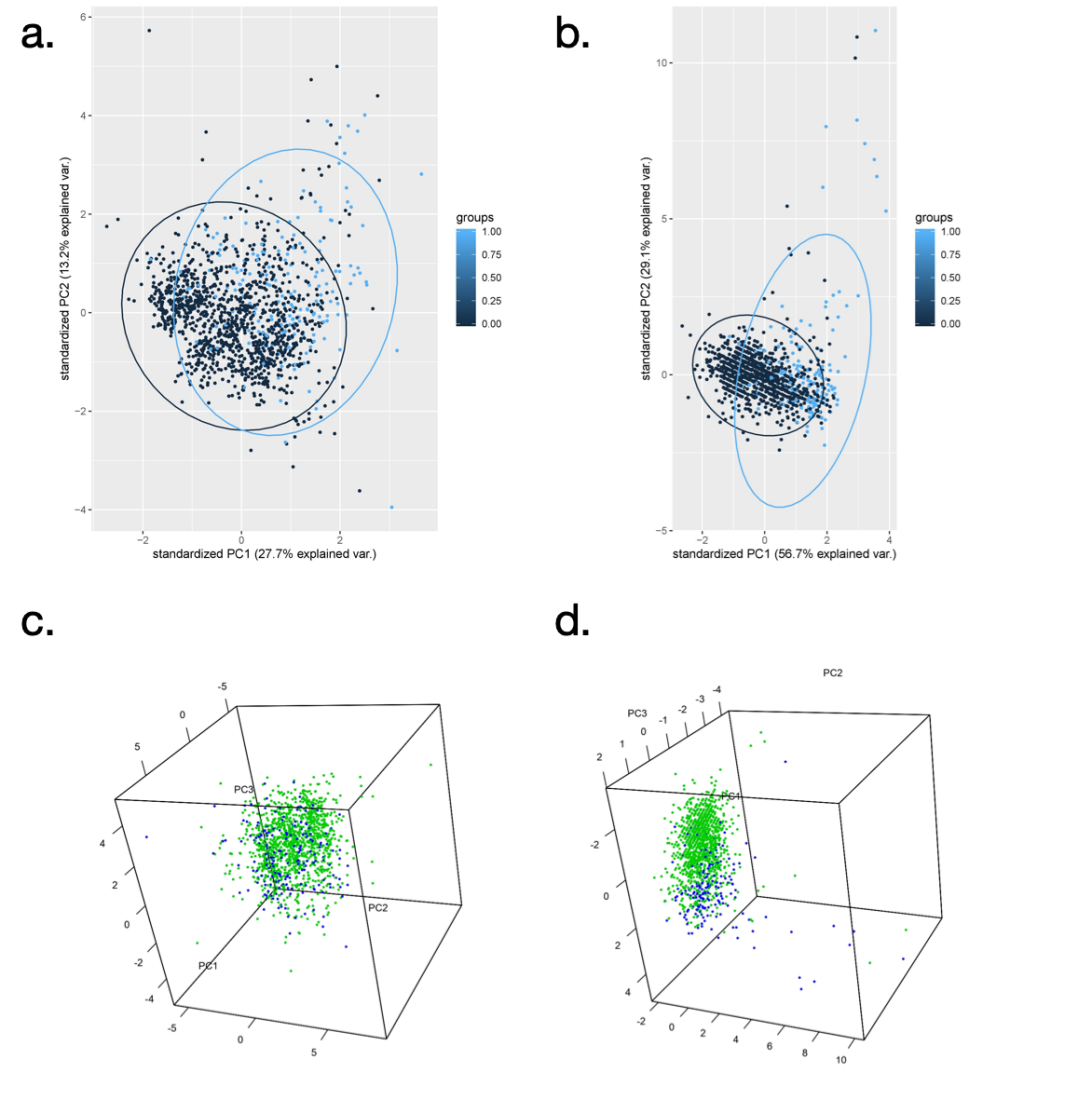
Principal components analysis (PCA) of metabolic and nonmetabolic groups by two-dimensional and three-dimensional visualization. (a) PCA with 95% CI shown as ellipses for all risk factors in Table 1. (b) PCA with 95% CI shown as ellipses for specific major variables in Table 3. Light blue nodes represent people diagnosed with metabolic syndrome, and dark blue nodes represent people without metabolic syndrome. PC1 explains the most variability among the samples, followed by PC2, PC3, and so on. (c) Three-dimensional PCA including PC1, PC2, and PC3 for all risk factor data from Table 1. (d) Three-dimensional PCA for specific major variables from Table 3.

The visualization of the two groups was achieved by PCA with the advantage of dimensionality reduction (Figure 2). All factors with significant outcomes by the tests mentioned above and shown in Table 1 depicted an intermixing view because the two groups of patients overlapped (Figure 2), with weak explanatory power for the first two principal components PC1 and PC2 at 27.7% and 13.2%, respectively. A variety of views in three-dimensional PCA plots are also displayed in Multimedia Appendix 1. The two groups could not be clearly discriminated, even if the coordinates were rotated in the three-dimensional graph.

**Table 1:** Descriptive statistics and testing of risk factors in health examination data with potential metabolic syndrome as the dependent variable.

Risk factors		No metabolic syndrome (N=1140)	Metabolic syndrome (N=193)	*P* value
**Chronic kidney disease^a^, n (%)**				
	Stage 1	585 (51.32)	71 (36.8)	.001
	Stage 2	530 (46.49)	115 (59.6)	
	Stage 3	24 (2.11)	6 (3.1)	
	Stage 4	1 (0.09)	1 (0.5)	
**Sex, n (%)**				
	female	564 (49.47)	44 (22.8)	<.001
	male	576 (50.53)	149 (77.2)	
**Obesity^a^, n (%)**				
	underweight	49 (4.30)	0 (0.0)	<.001
	normal weight	667 (58.51)	22 (11.4)	
	overweight I	293 (25.70)	65 (33.7)	
	overweight II	131 (11.49)	106 (54.9)	
Age (years), median (IQR)		44 (38-50)	45 (40-51)	.12
**Hepatic indices, median (IQR)**		4.6 (4.4-4.8)	4.7 (4.5-4.8)	<.001
	Albumin (g/dL)	2.26 (1.637-3.12)	2.43 (1.71-3.14)	.15
	AFP^b^ (ng/mL)	58 (48-69)	62 (54-75)	<.001
	ALKp^c^ (IU/L)	20 (17-24)	24 (19-31)	<.001
	GOT^d^ (IU/L)	19 (13-27)	33 (22-51)	<.001
	GPT^e^ (IU/L)	0.6 (0.5-0.8)	0.7 (0.5-0.9)	.08
	Total bilirubin (mg/dL)	0.2 (0.2-0.3)	0.2087 (0.2-0.3)	.66
	Direct bilirubin (mg/dL)	16 (12-25)	29 (20-45)	<.001
	γ-GT^f^ (U/L)	239 (209-274)	311 (271-340)	<.001
	CAP^g^ score (dB/m)	4 (3.4-4.8)	4.9 (4.3-5.8)	<.001
	E score (kPa)	12 (10-15)	13 (11-15)	.03
**Nephritic indices, median (IQR)**				
	BUN^h^ (mg/dL)			
	Creatinine (mg/dL)	0.8 (0.6-0.9)	0.9 (0.7-1.0)	<.001
	MDRD^i^	91.07 (81.3-105.17)	86.23 (75.05-98.82)	<.001
	UA^j^ (mg/dL)	5.2 (4.3-6.5)	6.3 (5.5-7.3)	<.001
**Blood lipid and thyroid markers, median (IQR)**				
	Cholesterol (mg/dL)	187 (165-208)	194 (165-220)	.03
	LDL^k^ (mg/dL)	121 (101-142)	136 (106-158)	<.001
	HbA_1c_^l^ (%)	5.4 (5.2-5.7)	5.7 (5.4-6.1)	<.001
	TSH^m^ (μIU/mL)	1.93 (1.30-2.52)	1.89 (1.38-2.51)	.83

^a^Progressive discrete variables.

^b^AFP: alpha-fetoprotein.

^c^ALKp: alkaline phosphatase.

^d^GOT: glutamic-oxalocetic transaminase.

^e^GPT: glutamic-pyruvic transaminase.

^f^γ-GT: gamma-glutamyl transpeptidase.

^g^CAP: controlled attenuation parameter.

^h^BUN: blood urea nitrogen.

^i^MDRD: Modification of Diet in Renal Disease.

^j^UA: uric acid.

^k^LDL: low-density lipoprotein cholesterol.

^l^HbA_1c_: glycated hemoglobin.

^m^TSH: thyroid-stimulating hormone.

Next, we applied multivariate logistic regression to assess factors influencing metabolic syndrome. As shown in Table 2, the number of significant variables was reduced to 3, and included obesity, CAP score, and HbA_1c_. Among these, HbA_1c_ was obtained from blood tests, whereas information on obesity and the CAP score was obtained through noninvasive means. Notably, obesity and HbA_1c_ exhibited high odds ratios, exceeding 2. In addition, the variance inflation factor was taken into account for multicollinearity.

**Table 2:** Multivariate logistic regression analysis of risk factors related to metabolic syndrome.

Factor	Odds ratio^a^ (95% CI)	VIF^b^	ΔVIF^c^	*P* value
Sex, Male/Female	0.742 (0.335-1.641)	3.590	1.630	.99
Age, years	1.025 (0.999-1.051)	1.622	1.506	.15
Obesity	2.915 (2.175-3.907)	1.429	1.406	<.001
Albumin, g/dL	1.866 (0.821-4.239)	1.218	1.176	.12
AFP^d^, ng/mL	1.045 (0.915-1.193)	1.162	1.153	.48
ALKp^e^, IU/L	0.995 (0.983-1.007)	1.158	1.140	.52
GOT^f^, IU/L	0.959 (0.923-0.997)	*7.226*	-	-
GPT^g^, IU/L	1.023 (1.003-1.045)	*7.747*	*1.555*	.51
Total bilirubin, mg/dL	2.599 (0.562-12.015)	*8.334*	*1.246*	.39
Direct bilirubin, mg/dL	0.011 (0-2.507)	*8.413*	-	-
γ-GT^h^, U/L	1.002 (0.994-1.009)	1.414	1.379	.77
CAP^i^ score, dB/m	1.011 (1.007-1.016)	1.455	1.398	<.001
E score, kPa	1.046 (0.926-1.182)	1.284	1.256	.61
CKD^j^	1.135 (0.615-2.097)	3.387	2.550	.27
BUN^k^, mg/dL	0.952 (0.893-1.016)	1.397	1.338	.13
Creatinine, mg/dL	4.288 (0.196-94.014)	*10.957*	-	-
MDRD^l^	1.012 (0.994-1.031)	4.860	2.863	.39
UA^m^, mg/dL	1.127 (0.967-1.314)	1.642	1.596	.08
Cholesterol, mg/dL	1.003 (0.986-1.021)	*9.855*	-	-
LDL^n^ mg/dL	0.994 (0.976-1.012)	*9.701*	*1.069*	.82
HbA_1c_^o^, %	2.170 (1.631-2.888)	1.236	1.230	<.001
TSH^p^, μIU/mL	0.876 (0.727-1.054)	1.086	1.078	.13

^a^The odds ratio represents the exp(β), which is the exponential of the estimator in logistic regression.

^b^VIF: variance inflation factor (to check multicollinearity); factors with high VIF values are italicized.

^c^ΔVIF: variance inflation factor after removal of predictor variables with high VIF values; VIF values with a sharp decline are italicized.

^d^AFP: alpha-fetoprotein.

^e^ALKp: alkaline phosphatase.

^f^GOT: glutamic-oxalocetic transaminase.

^g^GPT: glutamic-pyruvic transaminase.

^h^γ-GT: gamma-glutamyl transpeptidase.

^i^CAP: controlled attenuation parameter.

^j^CKD: chronic kidney disease.

^k^BUN: blood urea nitrogen.

^l^MDRD: Modification of Diet in Renal Disease.

^m^UA: uric acid.

^n^LDL: low-density lipoprotein cholesterol.

^o^HbA_1c_: glycated hemoglobin.

^p^TSH: thyroid-stimulating hormone.

**Figure 3:**
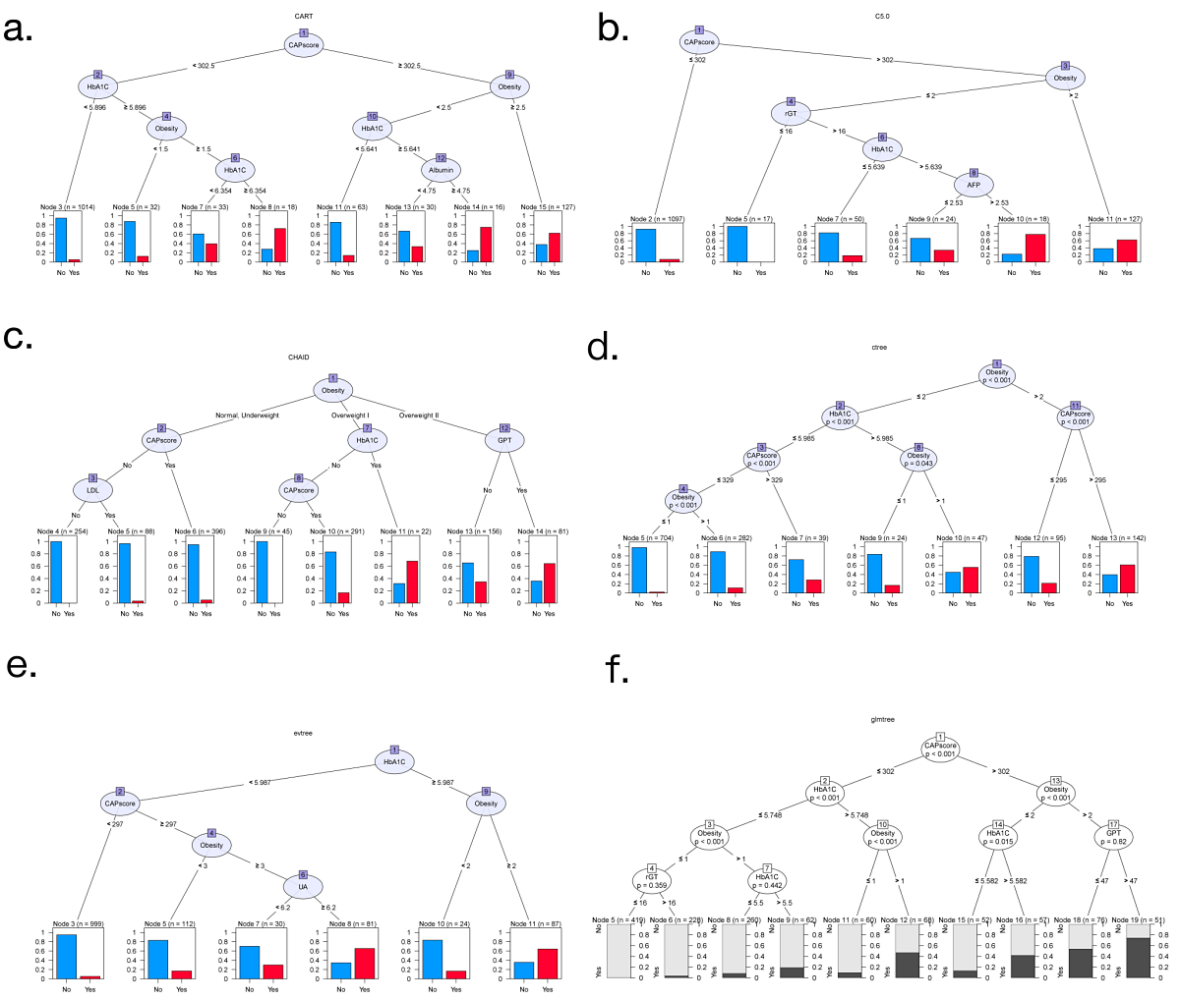
Metabolic syndrome prediction by various decision tree models. The decision tree takes on a flowchart-like structure. The six most commonly used decision trees are shown: (a) classification and regression tree (CART), (b) C5.0 classification tree modified from C4.5 and ID3 tree, (c) chi-square automatic interaction detection (CHAID), (d) conditional inference tree (ctree), (e) evolutionary learning of globally optimal tree (evtree), and (f) generalized linear model tree (glmtree). Each decision tree is applied for the prediction of metabolic syndrome to explore the factors with the greatest influence as an index to distinguish metabolic syndrome.

To inspect the potential indices used for metabolic syndrome, several types of decision trees were applied to health examination data for the classification of metabolic syndrome (Figure 3). In general, obesity, CAP score, and HbA_1c_ were found to be important predictive variables in the decision trees. Moreover, important variables appearing in each node of the decision trees were recorded 100 times (Table 3). CAP score, obesity, and HbA_1c_ were regarded as outstanding variables in the root, and E score, γ-GT, LDL, and GPT were secondary variables in the decision trees. The thresholds for factors classified as nodes are listed on the branches of each decision tree. In addition, a right skew pattern at the leaves was apparent and expected because the classification of metabolic syndrome was achieved efficiently and hierarchically by the decision trees (Figure 3).

**Table 3:** Major factors as classified nodes in decision trees^a^.

Decision tree	Root^b^	Primary node^c^ (root included)	Secondary node^d^	
CART^e^	*CAP* ^f^ *score (0.91)* *Obesity (0.09)*	*CAP score (0.99)* *HbA_1c_*^g^*(0.95)* *Obesity (0.94)* E score (0.05)^v^	*HbA_1c_ (0.93)* *Obesity (0.54)* γ-GT^h^ (0.40)^v^ Age (0.36) GOT^i^ (0.31) UA^j^ (0.28) AFP^k^ (0.25) E score (0.21)^v^	Total bilirubin (0.19) Albumin (0.18) ALKp^l^ (0.17) *CAP score (0.17)* GPT^m^ (0.17)^v^ LDL^n^ (0.15)^v^ MDRD^o^ (0.13) TSH^p^ (0.10)
C5.0	*CAP score (0.90)* *Obesity (0.06)* *HbA_1c_ (0.04)*	*Obesity (1.06)* *CAP score (0.94)* *HbA_1c_ (0.25)*	*HbA_1c_ (0.52)* Total bilirubin (0.41) GOT (0.35) γ-GT (0.34)^v^ E score (0.29)^v^	Sex (0.28) *Obesity (0.23)* AFP (0.19) CKD^q^ (0.16) UA (0.11)
CHAID^r^	*Obesity (1.00)*	*Obesity (1.00)* GPT (0.99)^v^ *HbA_1c_ (0.74)* *CAP score (0.70)* LDL (0.42)^v^ γ-GT (0.06)^v^	*CAP score (1.21)* GPT (0.41)^v^ E score (0.32)^v^ LDL (0.32)^v^ TSH (0.19) Sex (0.16)	AFP (0.12) *HbA_1c_ (0.12)*
ctree^s^	*Obesity (0.96)* *CAP score (0.04)*	*HbA_1c_ (0.89)* *CAP score (0.85)* *Obesity (0.51)* γ-GT (0.14)^v^	*Obesity (1.23)* *CAP score (0.67)* *HbA_1c_ (0.66)* LDL (0.17)^v^	AFP (0.16)
evtree^t^	*Obesity (0.33)* HbA_1c_ (0.30) *CAP score (0.17)* Escore (0.05)^v^ UA (0.05)	*Obesity (0.82)* *HbA_1c_ (0.58)* *CAP score (0.53)* Escore (0.10)^v^ UA (0.10) rGT (0.05)^v^	*HbA_1c_ (0.48)* *CAP score (0.44)* *Obesity (0.42)* UA (0.26) E score (0.25)^v^ ALKp (0.19)	GOT (0.19) γ-GT (0.13)^v^ GPT (0.11)^v^
glmtree^u^	*CAP score (0.85)* *Obesity (0.15)*	*Obesity (1.16)* *CAP score (0.92)* *HbA_1c_ (0.79)* LDL (0.06)^v^	*Obesity (1.13)* *HbA_1c_ (0.85)* γ-GT (0.79)^v^ Escore (0.35)^v^ GPT (0.26)^v^	LDL (0.23)^v^ *CAP score (0.22)* MDRD (0.18) GOT (0.15)

^a^Major variables are listed with their weights as candidate nodes in each decision tree; since some variables may be considered candidate nodes in the decision tree more than once, the proportion of variables can be larger than 1.

^b^The root shows factors appearing as the first classified node and their proportions.

^c^The primary node (italicized) includes variables selected as the top three nodes (root included) with their proportions (>0.05); variables with lower weights as candidate nodes in the primary nodes are excluded.

^d^The secondary node includes all remaining candidate nodes in each decision tree with their proportions; only candidate nodes with proportions >0.1 with a certain influence in the classification of metabolic syndrome are shown.

^e^CART: classification and regression trees.

^f^CAP: controlled attenuation parameter.

^g^HbA_1c_: glycated hemoglobin.

^h^γ-GT: gamma-glutamyl transpeptidase.

^i^GOT: glutamic-oxalocetic transaminase.

^j^UA: uric acid.

^k^AFP: alpha-fetoprotein.

^l^ALKp: alkaline phosphatase.

^m^GPT: glutamic-pyruvic transaminase.

^n^LDL: low-density lipoprotein cholesterol.

^o^MDRD: Modification of Diet in Renal Disease.

^p^TSH: thyroid-stimulating hormone.

^q^CKD: chronic kidney disease.

^r^CHAID: chi-square automatic interaction detection.

^s^ctree: conditional inference tree.

^t^evtree: evolutionary learning of globally optimal tree.

^u^glmtree: generalized linear model tree.

^v^Secondary variables for classification of metabolic syndrome in several decision tree algorithms.

PCA was then applied again to visualize the nonmetabolic syndrome and metabolic syndrome groups according to the prominence of factors from the decision trees, which comprised the CAP score, obesity, and HbA_1c_ (Figure 2b). PC1 and PC2 explained greater variability of 56.7% and 29.1%, respectively. With this analysis, discrimination between the two groups was evident, although the junction of the two groups was explicit in the union (Multimedia Appendix 2).

**Figure 4:**
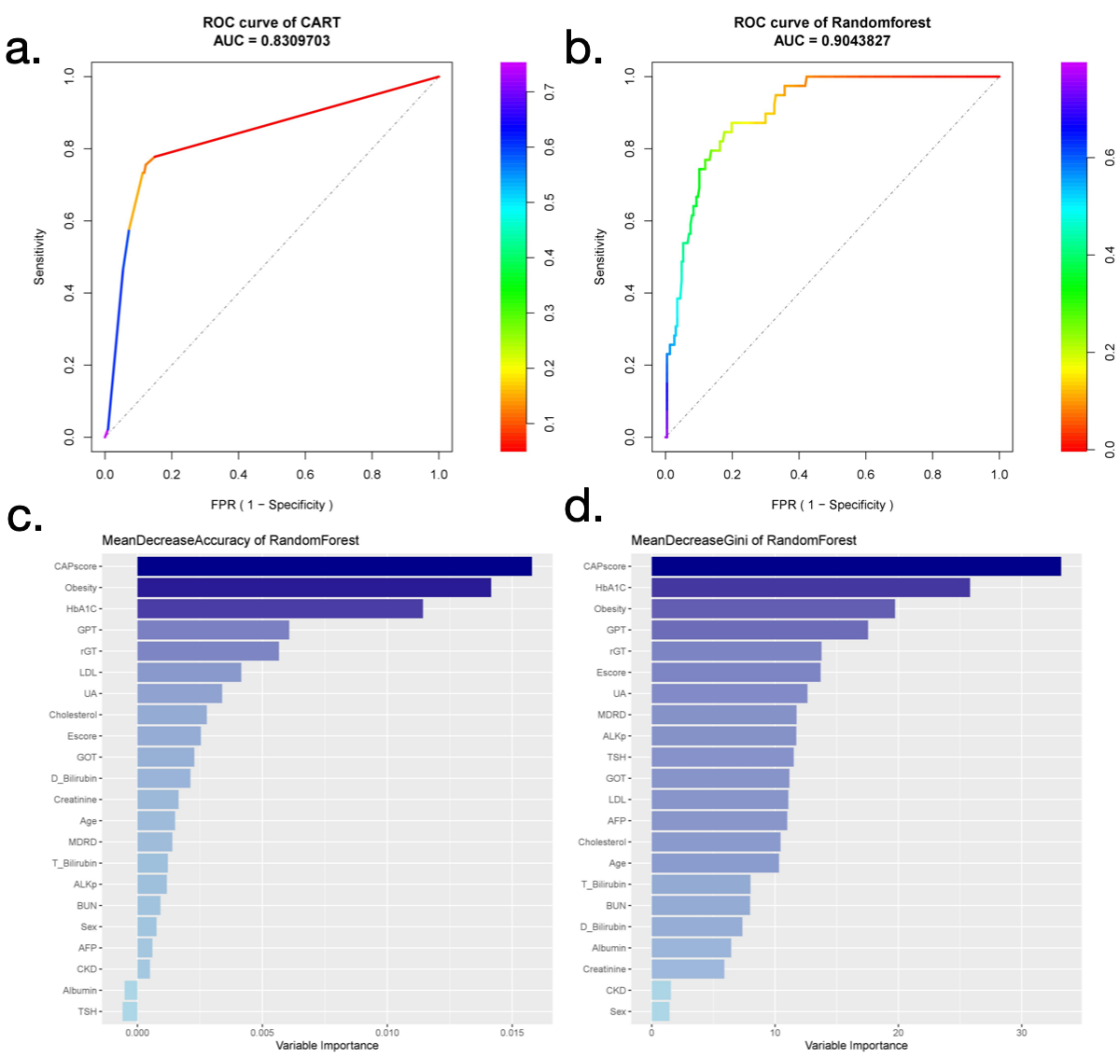
Random forest model for predicting classification performance and variable importance. Receiver operating characteristic (ROC) curve with area under the curve values for (a) classification and regression tree and (b) random forest. The color bar indicates the value of specificity in the false positive rate. (c) Variable importance ordered by accuracy of a mean decrease in random forest. (d) Variable importance ordered by the gini index of a mean decrease in random forest. The leading variables obtained by random forest are listed in darker blue, and less important variables are in lighter blue.

Finally, the accuracies of various decision trees were determined using 500 rounds of random sampling from the entire health examination dataset with fixed-size divisions of training and testing sets (Table 4). Independent training and testing sets were used for each evaluation to confirm the performance and reliability of each model. The AUC of the ROC curve was determined to evaluate the performance of each decision tree and random forest (Table 4, Figure 4, and Multimedia Appendix 3). Prominent variables obtained with random forest are shown in Figure 4. In general, CAP score, obesity, HbA_1c_, GPT, and γ-GT were the leading variables in accuracy, whereas CAP score, HbA_1c_, obesity, GPT, γ-GT, and E score played essential roles in random forest for classification.

**Table 4:** Accuracya and area under the curve (AUC) values of various decision trees in receiver operating characteristic curve analysis.

Decision tree	Accuracy			F1-score				AUC
	minimum	mean	maximum		minimum	mean	maximum	
CART^b^	0.797	0.857	0.914		0.888	0.919	0.948	0.831
C5.0	0.805	0.861	0.921		0.884	0.922	0.951	0.769
CHAID^c^	0.823	0.873	0.917		0.894	0.930	0.956	0.867
ctree^d^	0.801	0.864	0.914		0.883	0.923	0.954	0.896
evtree^e^	0.805	0.857	0.906		0.880	0.920	0.953	0.815
glmtree^f^	–^g^	–	–		–	–	–	0.889
Random forest	0.812	0.870	0.940		0.888	0.928	0.959	0.904

^a^Accuracy and F1-score were calculated from 500 machine learning trials with different training sets for comparison with the number of candidate trees from random forest. Accuracy is the probability of true positives and true negatives for all data, whereas F1-score is a measure of performance, which is the harmonic mean of precision and recall. The dataset was divided 80% as the training set and 20% as the testing set independently for each analysis with randomized sampling.

^b^CART: classification and regression trees.

^c^CHAID: chi-square automatic interaction detection.

^d^ctree: conditional inference tree.

^e^evtree: evolutionary learning of globally optimal tree.

^f^glmtree: generalized linear model tree.

^g^The terminal nodes of the R package glmtree are not a simple classification form to calculate the confusion matrix for accuracy; therefore, the area under the curve was used to reach a balance in comparison between the seven decision tree techniques on the same training and testing set.

## Discussion

### Principal Findings

The use of artificial intelligence in health care, particularly machine learning methods, can help to discover underlying patterns and correlations through the learning of data-driven prediction models. We applied various machine learning techniques to visualize and investigate predictive variables leading to metabolic syndrome, which revealed that obesity, serum GOT, serum GPT, CAP score, and HbA_1c_ are the most important predictive variables.

Among these predictive variables, the predictive power of the CAP score was similar to that of other key indices such as obesity. Despite the significance of the CAP score, these factors make sense cumulatively rather than as exclusive alternatives. In other words, more research is required to determine whether the CAP score can be used as a standalone test method to screen for metabolic syndrome, and whether a minimum set of nonblood test variables can be combined with the CAP score to improve the accuracy of predicting metabolic syndrome. Such future research may help subjects who are resistant to the inconvenience of overnight starvation or painful blood assays.

Metabolic syndrome demonstrates a spectrum of physiological manifestations with groups of pathologies that are complicated and progressive. Traditional diagnostic criteria often dichotomize the population into those with metabolic syndrome and those without. However, based on the results of our PCA, such a sharp distinction may be inappropriate. We found that CAP score, obesity, and HbA_1c_ were the principal factors predicting metabolic syndrome, although E score, γ-GT, LDL, and GPT also considerably affected the predictions. Notably, GPT had more predictive power than GOT. We consider this difference to be related to aspartate aminotransferase as a relatively less specific indicator of liver damage than alanine aminotransferase, which is common in patients with fatty liver. Our study suggests that current diagnostic criteria for metabolic syndrome fail to capture its wide range of presentations, and should thus be expanded to include hepatic and nephritic indices.

Liver-related indices such as γ-GT, GPT, and E score ranked among the highest predictors in our models. A previous study also showed a strong correlation between liver function tests and metabolic syndrome based on Pearson correlation coefficients [66]. HbA_1c_ is reported to be more closely associated with several chronic diseases than fasting plasma glucose. In addition, although fasting glucose levels are commonly believed to be reproducible across days, acute perturbations of glucose homeostasis due to stress and other factors have been described. By contrast, HbA_1c_ is not influenced by acute perturbations or insufficient fasting; thus, it can be measured at any time. Accordingly, HbA_1c_ might prove to be a more suitable predictor of metabolic syndrome [67].

Multivariate logistic regression has been extensively utilized in medical research, and its many biases have been well documented. One of the drawbacks we observed in our models was the multicollinearity problem. To avoid multicollinearity (Table 2), GOT, direct bilirubin, creatinine, and cholesterol were eliminated from the regression model. By contrast, the decision trees had few such disadvantages and offered more intuitive visualizations. The trained decision tree models could also be more easily interpreted by human experts, which is vital for establishing various important pathways to metabolic syndrome. In general, our result that random forest has the best accuracy in detecting metabolic syndrome agrees with previous research [68]. One of the reasons for the better accuracy of a random forest model is that it creates multiple decision trees and then combines the output generated by each tree; each tree is built from a sample drawn with replacement from the training set. This approach therefore removes the bias that a decision tree model might introduce in the system, thus substantially improving the predictive power.

### Limitations

This study has several limitations. First, this was a retrospective study, and therefore a sufficiently powered prospective cohort study is needed to conclusively address the usefulness of supervised machine learning models to diagnose metabolic syndrome. Second, this study included only health-conscious Taiwanese participants that underwent a self-paid health examination; therefore, this study should be replicated and validated in other populations. Third, this study failed to include some new obesity biomarkers (such as leptin and adiponectin) that may further improve the prediction of metabolic syndrome [69].

### Conclusion

To the best of our knowledge, this is the first study to apply machine learning algorithms to identify metabolic syndrome in subjects examined with FibroScan. We found that decision tree learning algorithms identified metabolic syndrome in self-paid health examination subjects with high accuracy, and obesity, serum GOT, serum GPT, CAP score, and HbA_1c_ emerged as important predictive variables. More research is required to validate the CAP score as a standalone test method to screen for metabolic syndrome, and to determine whether a minimum set of nonblood tests variables can be combined with the CAP score to improve the accuracy of predicting metabolic syndrome.

## Appendix

Multimedia Appendix 1 Three-dimensional principal components analysis (PCA) of all risk factors. The three-dimensional PCA plots provide different visual points to observe the scatter of both the metabolic syndrome and nonmetabolic syndrome groups. All factors in Table 1 are considered in this analysis. The leading principal components PC1, PC2, and PC3—which explain more variability among the samples—are shown in all three-dimensional graphs. The aggregation of two groups is obvious when rotating the coordinate in the three-dimensional graph.

Multimedia Appendix 2 Three-dimensional principal components analysis of only the major risk factors. In this case, the distinction between the metabolic syndrome and nonmetabolic syndrome groups is apparent because only the major variables obtained from Table 3 are included, although the borders of the two groups still overlap.

Multimedia Appendix 3 Receiving operator characteristic curves and area under the curve (AUC) values of six decision trees. The specificity is revealed by the color bar, and the diagonal line is presented as a dashed line. Most AUC values exceed 0.80 except for that of the C5.0 tree.

## Abbreviations

AFP: alpha-fetoprotein
ALKp: alkaline phosphatase
AUC: area under the curve
BUN: blood urea nitrogen
CAP score: controlled attenuation parameter score
CART: classification and regression tree
CHAID: Chi-square automatic interaction detection
CKD: chronic kidney disease
ctree: conditional interference tree
eGFR: estimated glomerular filtration rate
E score: liver stiffness score
evtree: evolutionary learning of globally optimal trees
γ-GT: gamma-glutamyl transpeptidase
GOT: serum glutamic oxaloacetic transaminase
GPT: serum glutamic pyruvic transaminase
HbA_1c_: glycated hemoglobin
HDL: high-density lipoprotein
LDL: low-density lipoprotein
MDRD: Modification of Diet in Renal Disease
NAFLD: nonalcoholic fatty liver disease
PCA: principal components analysis
ROC: receiver operating characteristic
TSH: thyroid-stimulating hormone
UA: uric acid
VIF: variance inflation factor
