# Supplementary figures and images for: Predicting Metabolic Syndrome With Machine Learning Models Using a Decision Tree Algorithm: Retrospective Cohort Study
Source: JMIR Med Inform. 2020 Mar 23;8(3):e17110. doi: 10.2196/17110 (PMC7136841; doi:10.2196/17110)

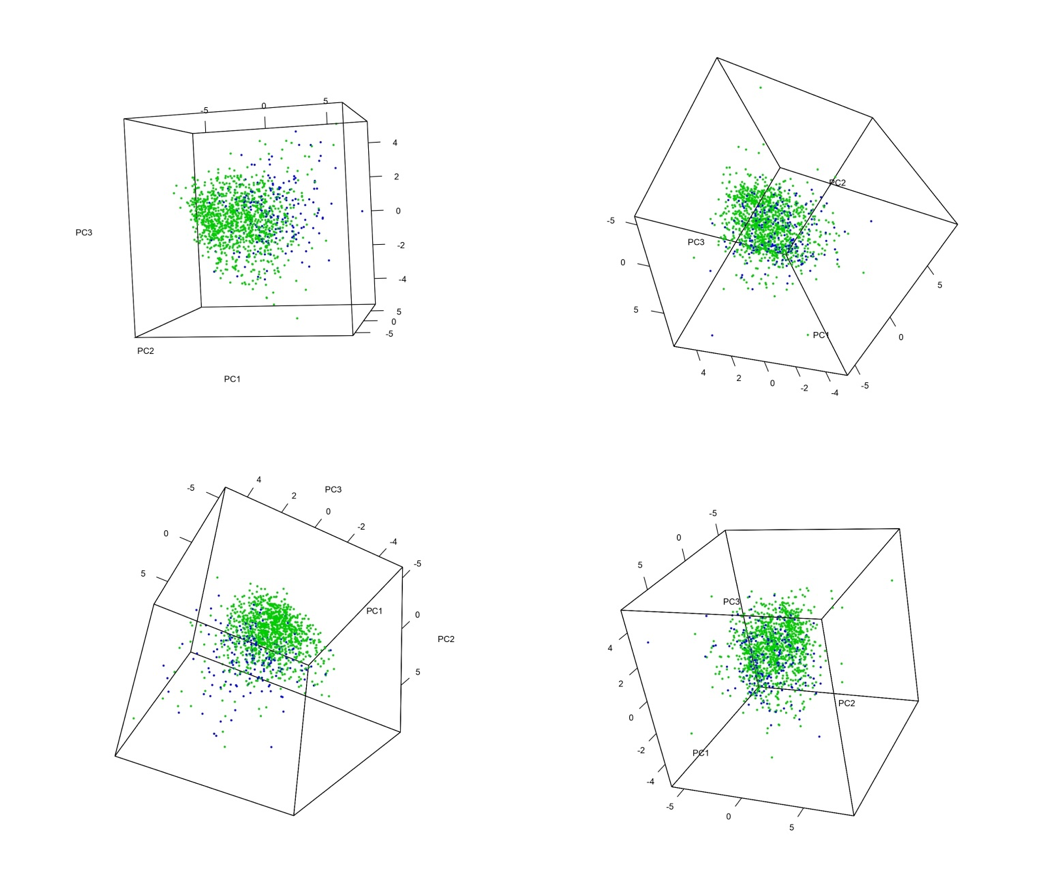

Supplement: Multimedia Appendix 1 [file medinform_v8i3e17110_app1.png]

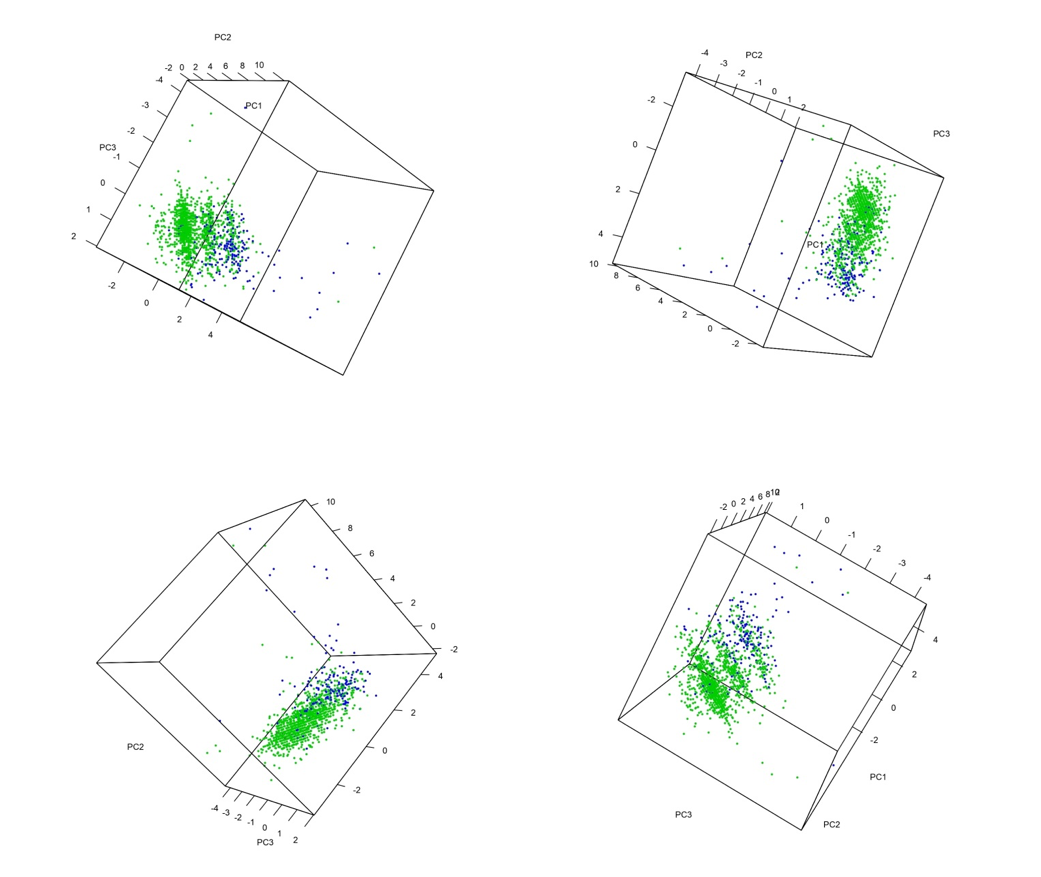

Supplement: Multimedia Appendix 2 [file medinform_v8i3e17110_app2.png]

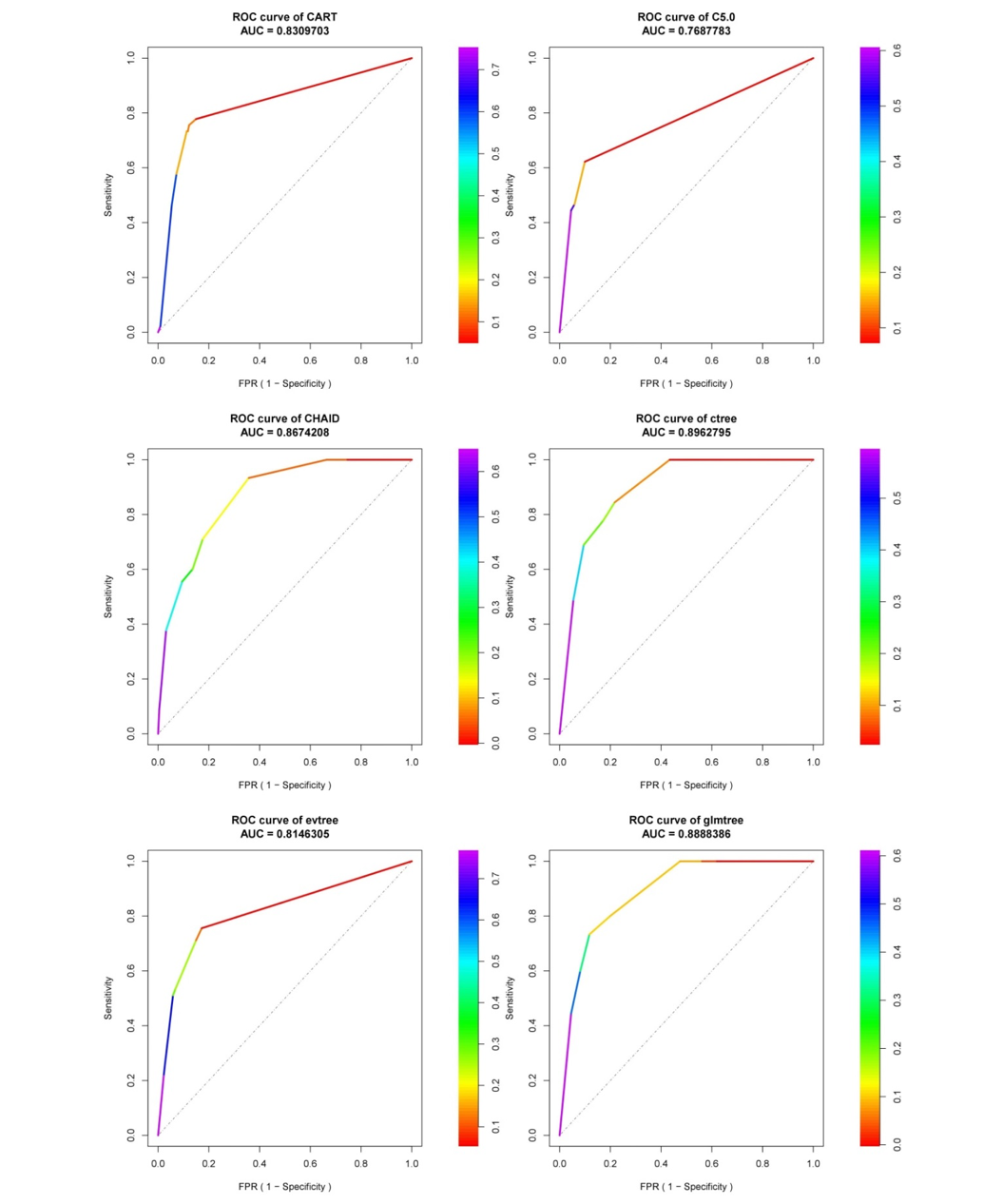

Supplement: Multimedia Appendix 3 [file medinform_v8i3e17110_app3.png]
